# Supplementary figures and images for: Qualitative evidence synthesis of values and preferences to inform infant feeding in the context of non-HIV transmission risk
Source: PLoS One. 2020 Dec 1;15(12):e0242669. doi: 10.1371/journal.pone.0242669 (PMC7707527; doi:10.1371/journal.pone.0242669)

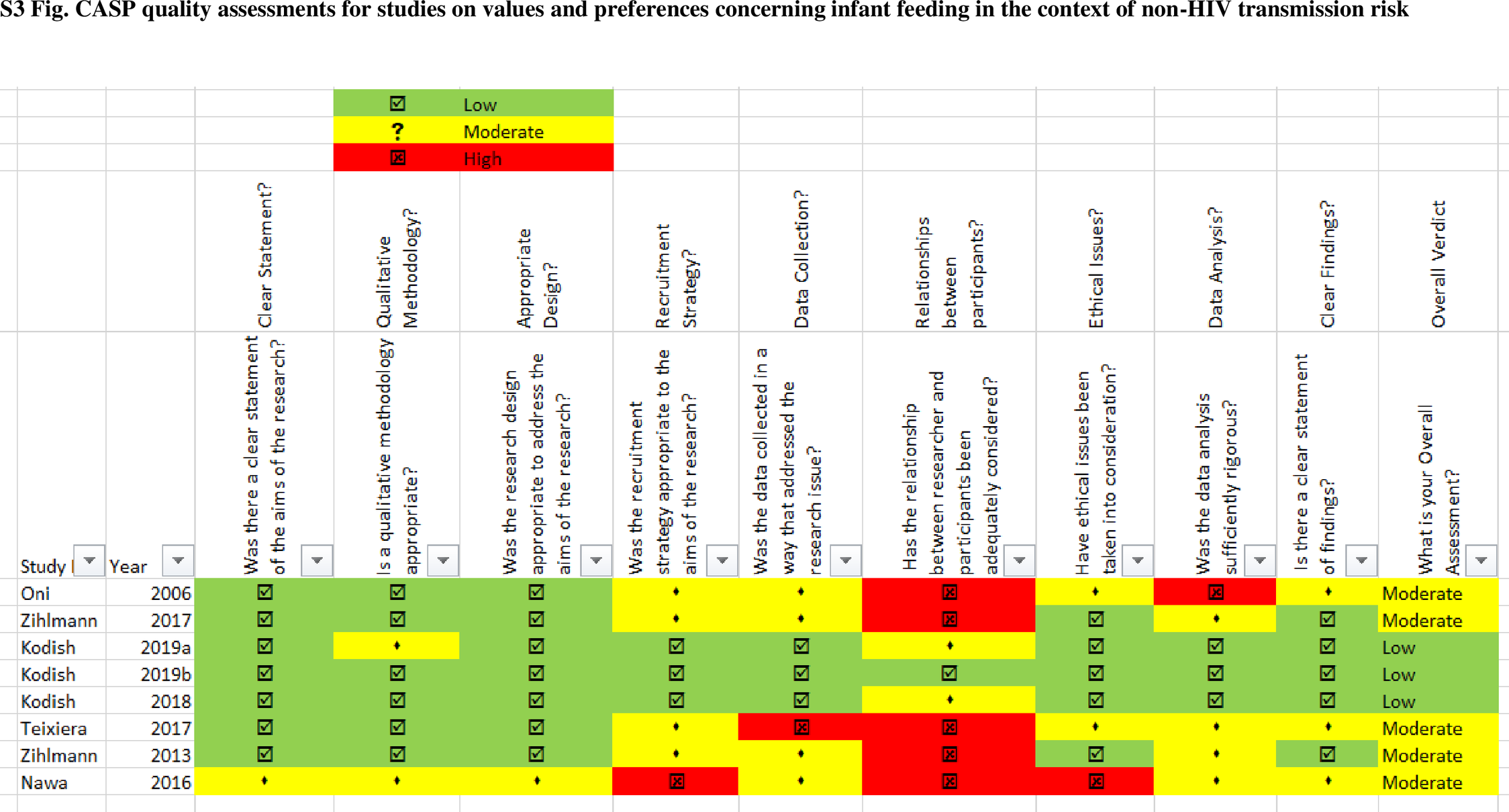

Supplement: S1 Fig — (TIF) [file pone.0242669.s002.tif]
