# Supplementary material for: Qualitative evidence synthesis of values and preferences to inform infant feeding in the context of non-HIV transmission risk
Source: PLoS One. 2020 Dec 1;15(12):e0242669. doi: 10.1371/journal.pone.0242669 (PMC7707527; doi:10.1371/journal.pone.0242669)
Supplement: S1 Table — (DOCX) [file pone.0242669.s003.docx]

**S1 Table. MEDLINE search**

| **Review Question** | **What are the views of pregnant women, mothers, family members and health practitioners and providers (midwives) concerning infant feeding (including breast-feeding and its alternatives) when there is a risk of potentially transmissible illness?** | | | | | | | |
| --- | --- | --- | --- | --- | --- | --- | --- | --- |
| **Key Concepts** | **Infant Feeding** | **AND** | **Infection** | **AND** | **(Perspectives** | **OR** | **Qualitative)** |  |
| **Synonyms** | (MH "Infant Formula") OR  (MH "Milk, Human+") OR (MH "Milk Expression") OR (MH "Lactation") OR  (MH "Infant Feeding+") OR  (MH "Bottle Feeding") OR  (MH "Breast Feeding+") |  | "vaccination" OR  (MH "Immunization+") OR "option b+" OR (MH "HIV Infections+") OR  (MH "Disease Transmission, Vertical") OR  (MH "Communicable Diseases+") OR (MH Infection+") |  | perception$ or attitude$ or perspective$ or opinion$ or experience$ or view$ |  | (MH "Qualitative Studies+") OR (MH "Multimethod Studies") OR "qualitative" |  |
