# Supplementary material for: Qualitative evidence synthesis of values and preferences to inform infant feeding in the context of non-HIV transmission risk
Source: PLoS One. 2020 Dec 1;15(12):e0242669. doi: 10.1371/journal.pone.0242669 (PMC7707527; doi:10.1371/journal.pone.0242669)
Supplement: S2 Table — (DOCX) [file pone.0242669.s004.docx]

**S2** **Table. Excluded studies (full-text checked) [principal reason for exclusion, other than HIV/AIDS]:**

| Author, year | Title, journal |
| --- | --- |
| Qualitative studies but no infant feeding and transmission risk data | |
| Blasco Hernandez 2016 | Knowledge and experiences of Chagas disease in Bolivian women living in Spain: A qualitative study, Global Health Action, 9(1). |
| Cassidy 2019 | Milk Banking and the Uncertain Interaction Between Maternal Milk and Ethanol (Muimme), Breastfeeding Medicine, 13(7): P-115. |
| Da Sa 2017 | Parental Needs in the Care for Children With Zika Virus-Induced Microcephaly, Revista Brasileira em Prom da Sáude. |
| Dynes 2015 | Perceptions of the Risk for Ebola and Health Facility Use Among Health Workers and Pregnant and Lactating Women — Kenema District, Sierra Leone, September 2014, Morbidity and Mortality Weekly Report, 63 / Nos. 51 & 52: 1226-1227. |
| Miracle 2004 | Mothers’ Decisions to Change From Formula to Mothers’ Milk for Very-Low-Birth-Weight Infants, JOGNN Clinical Research, 33 (6): 692-703. |
| Tembo 2015 | Exclusive Breast Feeding Practice in Zambia, Medical Journal of Zambia, Vol. 42, No. 3: 124-129 |
| Teixiera 2013 | Manuseio Com Massa De Modelar: Uma Estratégia Sensível De Coleta De Dados Na Pesquisa Em Saúde E Enfermagem, Texto Contexto Enferm, Florianópolis, 2013 Jul-Set; 22(3): 857. |
| Quantitative studies | |
| Krans 2018 | Hepatitis C Virus Knowledge Among Pregnant Women with Opioid Use Disorder, Maternal and Child Health Journal, 22:1208–1216. |
