# Supplementary material for: Qualitative evidence synthesis of values and preferences to inform infant feeding in the context of non-HIV transmission risk
Source: PLoS One. 2020 Dec 1;15(12):e0242669. doi: 10.1371/journal.pone.0242669 (PMC7707527; doi:10.1371/journal.pone.0242669)
Supplement: S3 Table — (DOCX) [file pone.0242669.s005.docx]

**S3 Table. GRADE-CERQual Evidence profile: Factors relating to individuals**

| **Summary of review finding** | **Studies contributing to the review finding** | **Methodological limitations** | **Coherence** | **Adequacy** | **Relevance** | ***GRADE-CERQual assessment of confidence in the evidence*** | **Explanation of GRADE-CERQual assessment** |
| --- | --- | --- | --- | --- | --- | --- | --- |
| Lactating women and frontline workers lack knowledge about risk of transmission from mother-to-child (MTC) by breast-feeding | (37,42,43) | Two studies with moderate or serious methodological limitations because of their recruitment strategies, lack of reflexivity, and limited details on data collection and analysis. The appropriateness of the methodology was also questionable in one study. One study was considered high quality. | Moderate concerns about coherence because the link between the data are finding are explicit in one study but inferred in the second. One study was considered to have high levels of coherence. | Some concerns about adequacy given only a small amount of data in two of these studies. However, one sought data saturation and a comprehensive purposive sample. | Some concerns about relevance because there are only three studies, one in women with HTLV-1 only, one in a broad demographic, and the review question is not a focus of this second study, and one in the context of an Ebola outbreak in one country. | Low confidence | Three studies (French Guiana, Japan, Sierra Leone). There are some concerns about coherence, and serious concerns about methodological limitations, adequacy and relevance (one study was HTLV-1 and one on influenza vaccination) in two of the studies. One of the studies did not have methodological limitations. |
| New mothers were strongly influenced by the information and advice on MTC transmission provided by specialist health staff who have expertise in this condition.  New mothers feel empowered by this information and advice | (38,39,42) | Three studies, all with moderate concerns about methodological limitations because of their recruitment strategies, and limited details on data collection and analysis, and serious concerns about lack of reflexivity. | Minor concerns about coherence because the finding has clear support in the data. | Moderate concerns about adequacy because the data, though limited, are rich. | Moderate concerns about relevance because all three studies concern only a single potentially relevant condition, HTLV-1, and only from the perspective of the parents, but the finding is highly relevant to the review question. | Moderate confidence | Three studies (two Brazil, one French Guiana). There are moderate concerns about methodological limitations, coherence, adequacy and relevance (all studies only consider HTLV-1). |
| New mothers report that when information and advice is given by specialist health staff who have expertise in this condition, this gives them confidence in their choices | (38, 39) | Two studies, both with moderate concerns about methodological limitations because of their recruitment strategies, and limited details on data collection and analysis, and serious concerns about lack of reflexivity. | Minor concerns about coherence because the finding has clear support in the data. | Moderate concerns about adequacy because the data, though limited, are rich. | Moderate concerns about relevance because both studies concern only a single potentially relevant condition, HTLV-1, and only from the perspective of the parents, but the finding is highly relevant to the review question. | Moderate confidence | Two studies (both Brazil). There are moderate concerns about methodological limitations, coherence, adequacy and relevance (all studies only consider HTLV-1). |
| New mothers maintain strong expectations about the need to breastfeed if they are to form bonds with their baby | (36, 38-40) | Four studies with minor or moderate concerns about methodological limitations because of their recruitment strategies, and limited details on data collection and analysis. Three studies had serious concerns about lack of reflexivity. | Minor concerns about coherence because the finding has clear support in the data. | Moderate concerns about adequacy because four studies, but the data are limited. | Moderate concerns about relevance because the three studies only cover two potentially relevant conditions (HTLV-1 and Ebola) | Moderate confidence | Four studies (three Brazil, one Guinea). There are minor concerns over coherence, and moderate concerns about methodological limitations, relevance and adequacy. |
| Mothers experience stigma as a consequence of not being able to breastfeed | (37, 38, 39, 42) | Four studies, all with minor or moderate concerns about methodological limitations because of their recruitment strategies, and limited details on data collection and analysis, and serious concerns about lack of reflexivity. | Minor concerns about coherence because the finding has clear support in the data. | Moderate concerns about adequacy because the data, though limited, are rich. | Moderate concerns about relevance because three of the four studies concern only a single potentially relevant condition, HTLV-1, and only from the perspective of the parents, but the finding is highly relevant to the review question. | Moderate confidence | Four studies (two Brazil, one French Guiana, one Sierra Leone). There are moderate concerns about methodological limitations, coherence, adequacy and relevance (all studies only consider HTLV-1). |
| Mothers’ health can affect their ability to breastfeed | (36, 37, 41) | Three studies, two with minor concerns about methodological limitations, and one with serious limitations. | Moderate concerns in two of the studies about coherence because the link between the data and findings is largely implicit. | Serious concerns about adequacy because the data are very limited. | Serious concerns about relevance because two publications focus on a single condition (Ebola) and infant feeding is not a focus of these studies. | Low confidence | Three studies (Guinea, Sierra Leone, Brazil). There are moderate concerns about methodological limitations and coherence, and serious concerns over adequacy and relevance. |
