# Supplementary material for: Qualitative evidence synthesis of values and preferences to inform infant feeding in the context of non-HIV transmission risk
Source: PLoS One. 2020 Dec 1;15(12):e0242669. doi: 10.1371/journal.pone.0242669 (PMC7707527; doi:10.1371/journal.pone.0242669)
Supplement: S4 Table — (DOCX) [file pone.0242669.s006.docx]

**S4 Table. GRADE-CERQual Evidence profile: Community related factors**

| **Summary of review finding** | **Studies contributing to the review finding** | **Methodological limitations** | **Coherence** | **Adequacy** | **Relevance** | ***GRADE-CERQual assessment of confidence in the evidence*** | **Explanation of GRADE-CERQual assessment** |
| --- | --- | --- | --- | --- | --- | --- | --- |
| Health decision-makers and managers reported a prevalent attitude in the community that failure to breast-feed indicated contagion or infection | (38, 39) | Two studies, both with moderate concerns about methodological limitations because of their recruitment strategies, and limited details on data collection and analysis, and serious concerns about lack of reflexivity. | Minor concerns about coherence because the finding has clear support in the data. | Serious concerns about adequacy because there are only two studies and the data are very limited. | Serious concerns because both studies cover only one potentially relevant condition (HTLV-1), one country and one group: lactating mothers. | Low confidence | Two studies (both Brazil). There are minor concerns over coherence, moderate concerns about methodological limitations, but serious concerns about adequacy and relevance (e.g. HTLV-1 and Brazil only). |
| According to health decision-makers and managers, those in the community believed that alternatives to breast-feeding were not trustworthy | (40) | Minor concerns about methodological limitations because only moderate concerns about reflexivity. | Minor concerns about coherence because the finding has clear support in the data. | Serious concerns about adequacy of only one study with very limited data. | Serious concerns because the study covers only one potentially relevant condition (Ebola), and one group: health decision-makers and managers. | Low confidence | One study (Guinea and Sierra Leone) of a single condition (Ebola). There are minor concerns about methodological limitations and coherence, but serious concerns about adequacy and relevance. |
