# Supplementary material for: Qualitative evidence synthesis of values and preferences to inform infant feeding in the context of non-HIV transmission risk
Source: PLoS One. 2020 Dec 1;15(12):e0242669. doi: 10.1371/journal.pone.0242669 (PMC7707527; doi:10.1371/journal.pone.0242669)
Supplement: S5 Table — (DOCX) [file pone.0242669.s007.docx]

**S5 Table. GRADE-CERQual Evidence profile: Health system factors**

| **Summary of review finding** | **Studies contributing to the review finding** | **Methodological limitations** | **Coherence** | **Adequacy** | **Relevance** | ***GRADE-CERQual assessment of confidence in the evidence*** | **Explanation of GRADE-CERQual assessment** |
| --- | --- | --- | --- | --- | --- | --- | --- |
| Women and new mothers report a lack of knowledge among non-specialised health staff about certain conditions with a risk of MTC transmission by breast-feeding (e.g. HTLV-1) | (37, 38, 39, 40, 42) | Five studies, all with minor or moderate concerns about methodological limitations. Three studies have concerns because of their recruitment strategies, and limited details on data collection and analysis, and serious concerns about lack of reflexivity. | Minor concerns about coherence because the finding has clear support in the data. | Moderate concerns about adequacy because the data, though limited, are rich | Moderate concerns over relevance because the five studies concern only two conditions, HTLV-1 and Ebola, each only from the perspective of the parents or health providers, respectively. However, the finding is highly relevant to the review question. | Moderate confidence | Five studies (two Brazil, one each French Guiana, Sierra Leone, Guinea). There are minor concerns about coherence, and moderate concerns about methodological limitations, adequacy and relevance (all studies only consider HTLV-1 or Ebola, and only from a single perspective) |
| New mothers appreciate facilities that provide privacy for infant feeding because they are not exposed to observation by others and therefore are less likely to experience stigma from being identified as having a transmissible disease | (39) | Moderate concerns about methodological limitations because of the recruitment strategies, and limited details on data collection and analysis, and serious concerns about lack of reflexivity. | Minor concerns about coherence because the finding has clear support in the data. | Serious concerns about adequacy because the data are rich, but the finding is from a single study. | Serious concerns because the data are rich and highly relevant to the review question, but the finding is only from one potentially relevant condition (HTLV-1), and one group: lactating mothers. | Low confidence | One study (Brazil) with minor concerns about coherence, moderate concerns about methodological limitations, but serious concerns about adequacy and relevance (e.g. only HTLV-1) |
| Health decision-makers and managers report that establishing trust between providers and communities is important if established practices on infant feeding are to be successfully challenged when there is a disease outbreak (e.g. Ebola) | (37, 40, 41) | Three studies, two with minor concerns about methodological limitations because of moderate concerns about reflexivity. | Moderate concerns in two of the studies about coherence because the link between the data and findings is largely implicit | Serious concerns about adequacy because the data are very limited. | Serious concerns about relevance because the publications focus on a single condition (Ebola) and infant feeding is not a focus of the studies. | Low confidence | Three studies (Guinea, Sierra Leone). There are minor concerns about methodological limitations, moderate concerns about coherence, and serious concerns over adequacy and relevance. |
| Health decision-makers and managers report that it is important for alternatives to breast feeding to be available and trustworthy if established practices of exclusive breast feeding (EBF) are to be challenged | (37, 40) | Minor concerns about methodological limitations because only moderate concerns about reflexivity. | Moderate concerns about coherence because the link between the data and findings is largely implicit | Serious concerns about adequacy because the data are from two studies and are very limited. | Serious concerns about relevance because the studies focus on a single condition (Ebola) and infant feeding is not a focus of the study. | Low confidence | Two studies (Guinea and Sierra Leone) of a single condition (Ebola). There are minor concerns about methodological limitations, moderate concerns about coherence, and serious concerns over adequacy and relevance. |
