# Supplementary material for: Qualitative evidence synthesis of values and preferences to inform infant feeding in the context of non-HIV transmission risk
Source: PLoS One. 2020 Dec 1;15(12):e0242669. doi: 10.1371/journal.pone.0242669 (PMC7707527; doi:10.1371/journal.pone.0242669)
Supplement: S6 Table — (DOCX) [file pone.0242669.s008.docx]

**S6 Table. GRADE-CERQual Evidence profile: Socio-economic factors**

| **Summary of review finding** | **Studies contributing to the review finding** | **Methodological limitations** | **Coherence** | **Adequacy** | **Relevance** | ***GRADE-CERQual assessment of confidence in the evidence*** | **Explanation of GRADE-CERQual assessment** |
| --- | --- | --- | --- | --- | --- | --- | --- |
| Mothers report that the cost of alternatives to breast-milk can be prohibitive. | (37, 42) | Two studies, one with moderate concerns about methodological limitations because of the recruitment strategies, and limited details on data collection and analysis, and serious concerns about lack of reflexivity. | Minor concerns about coherence because the finding has clear support in the data. | Serious concerns about adequacy because there are only two studies and the data are very limited. | Serious concerns about relevance because one study covers only one potentially relevant condition (HTLV-1), country (French Guiana) and one group: lactating mothers. The second study explores the impact on nutrition and food availability during the Ebola outbreak (Sierra Leone) | Low confidence | Two studies (French Guiana, Sierra Leone) of two conditions (HTLV-1, Ebola). Minor concerns about coherence, moderate concerns about methodological limitations, but serious concerns about adequacy and relevance. |
