# Supplementary material for: Qualitative evidence synthesis of values and preferences to inform infant feeding in the context of non-HIV transmission risk
Source: PLoS One. 2020 Dec 1;15(12):e0242669. doi: 10.1371/journal.pone.0242669 (PMC7707527; doi:10.1371/journal.pone.0242669)
Supplement: Attachment — Submitted filename: Response to reviewers.docx [file pone.0242669.s009.docx]

**Response to reviewers**

| **Editor’s comments** | **Response** |
| --- | --- |
| Formatting | We hope that these issues have now all been addressed adequately. |
| Please update your search to include all studies published in the last 12 month. | We appreciate the importance of work being up-to-date, but would like to argue that further update searches are not necessary for this work at this point in time. We have added text (p.8) to clarify that the original search of the databases was performed in March 2019 (only last calendar year), and that update citation searches of included studies were being performed up to November 2019. The manuscript and its findings therefore fall within a 12-month period from the last update searches. |
| **Reviewer 1** | **Response** |
| The authors present a qualitative evidence synthesis to review and summarise current evidence on the values and preferences of pregnant women, mothers, family members, health practitioners, policy makers and providers regarding breast-feeding when there is a risk of transmitting an infectious disease from mother to infant. While there currently are few manuscripts available on this topic, some of which are of limited quality, the authors review, assess and summarise the findings adequately, resulting in adequate conclusions and recommendations. | Thank you – no action needed |
| - It is unclear why the job title is included in the affiliation of the last author. | Yes, this was an error – it has now been removed. |
| - The abbreviation MTC is introduced several times in the manuscript, yet not used consistently throughout the manuscript, e.g. abstract line 4, page 4/line 17, page 17 etc | Thank you – these have now all been revised to mother-to-child transmission (MTCT). |
| - Fig S3 is of poor quality and most of the text is not readable. Once updated, the manuscript might benefit from including the figure into the main text. | We have retained this as a supplementary file, but it is now S3 Fig and has been reformatted so that all of the text is visible and legible and the figure itself is much larger. |
| - Table 2 could be structured better to allow the reader to get a clearer picture of the information provided. Further there are some grammatical mistakes, e.g. the fullstops are missing in the perspectives column for study 43. | Thank you. The grammar has been checked and corrected, and the ‘Perspectives and sample characteristics’ column has been split into two separate columns to enhance readability. |
| - The sentence structure is unclear at some parts of the manuscript, e.g. p18/line 20-22 or p22/line 10-11 | These sentences and paragraphs have now been revised, thank you. |
| - It might be helpful to structure the result sections according to the infectious disease that was evaluated in the studies as values and preferences might differ depending on which infectious agent might be transmitted via breastfeeding | Thank you. The results narrative now presents the findings for each condition separately. |
| **Reviewer 2** | **Response** |
| The QES reports on an important area in the context of infant health. It also specifically addresses an area of breastfeeding and risks of MTC seldom studied compared to MTC transmission as it relates to HIV. The paper is well written and the methods adequately explained. The results are well synthesized and findings from the 8 studies well summarized. | Thank you – no action needed |
| page 2 line 14: replace breast feed with breastfeed | Thank you. This misspelling has now been corrected in this case and others. |
| page 2 line20: information such as? | Thank you – wording revised. |
| page 4 line 6: the sentence awkward - could be replaced with : Optimal breastfeeding is estimated to save more than 820 000 children.....? | Thank you – wording revised as suggested. |
| page 4 line 15: replace in with during. | Thank you – wording revised as suggested. |
| page 6 line 17: i suggest changing sentence to: could you change this to: To be included in the review and synthesis, studies were required to satisfy the criteria as outlined in Table 1. | Thank you – wording revised as suggested. |
| page 17 line 24: health staff such as who? in line 20 the use of health staff seem to refer to those trusted and not trusted and here only those not trusted. | Thank you – this has now been clarified; they are the ‘specialist’ health staff. |
| page 18 line 20: could this sentence be rephrased? | Thank you – wording revised. |
| page 19 line page 19 line 14: could this sentence be re-phrased? is this an attitude towards breastfeeding. This sentence is awkward.6-7: could not lactate or could not breastfeed? | Thank you. ‘Attitudes’ revised to ‘views’, and ‘lactate’ revised more accurately to ‘breastfeed’. |
